# Fish sauce fermentation using *Marinococcus halotolerans* SPQ isolate as a starter culture

**DOI:** 10.1002/fsn3.2024

**Published:** 2020-12-30

**Authors:** Anh Do Quynh Nguyen, Ashokkumar Sekar, Myoungjin Kim, Loc Phat Nguyen, Nga Thi Le, Sangjun Uh, Sukil Hong, Keun Kim

**Affiliations:** ^1^ R&D Department Masan Industrial One Member Co. Ltd Di An City Binh Duong province Vietnam; ^2^ Division of Bioindustry The University of Suwon Hwaseong Korea

**Keywords:** amino acid profile, fish sauce fermentation, histamine, *Marinococcus halotolerans*, starter culture, volatile compounds

## Abstract

A total of 344 halophilic bacteria were isolated from fish fermentation broths, solar salt crystals, seawater, and muds from ponds of salt pans in Vietnam and subjected to aroma evaluation using fish broth containing 29 ~ 30% (w/v) NaCl. One isolate from a salt crystal with the highest aroma score was selected, identified by using 16S rDNA sequence, and named *Marinococcus halotolerans* SPQ. The GC‐MS results of the fish broth fermented by *M. halotolerans* SPQ revealed elevated concentrations of several aroma compounds such as ethyl alcohol, 1‐propanol, 1‐butyl alcohol, 1‐amyl alcohol, and methionol. During the validation tests for *M. halotolerans* SPQ, using 2 kg of anchovy fish in 30% (w/v) NaCl at pH 5.78, the total and amino nitrogen values in the broth increased over time from 15.2 g/L at the beginning to 26.3 g/L at 6th month, with these values being comparable to those of the control. The ammoniacal nitrogen value (2.52 g/L) in the inoculated broth at 6th month was slightly higher than that (2.21 g/L) of control. The histamine content of the fish broth inoculated with *M. halotolerans* SPQ after 6 months was 110.12 mg/L, less than the maximum permitted safety limit of 200 mg/L, indicating it to be safe. Physical parameters, such as the total, amino, ammoniacal nitrogens, and histamine content of fish broth fermented by *M. halotolerans* MPQ met the standards for Vietnamese fish sauces. Two important umami amino acids, aspartic and glutamic acid, were seen to significantly increase, by 23.5% and 35.1%, respectively, even in the extremely harsh fermentation conditions posed by 30% (w/v) NaCl. The color, odor, and taste of the fish sauce fermented by *M. halotolerans* SPQ elicited the highest preference score accorded by the panelists. Taken together, *M. halotolerans* SPQ is a promising starter culture strain for fish sauce fermentation.

## Graphical abstract

Two important umami amino acids, aspartic and glutamic acid, were seen to significantly increase, by 23.5% and 35.1%, respectively, even in the extremely harsh fermentation conditions posed by 29.5% (w/v) NaCl at pH 5.78.

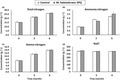

**Practical Applications**

The use of a starter culture of halophilic bacteria for fish sauce fermentation could improve the flavor of fish sauces and their consistency, with the additional benefit of enhanced productivity through a shortened fermentation process.

## 1 INTRODUCTION

Fish sauce is a popular condiment traditionally used in Asian countries for many years and which is now becoming more widely accepted in cuisine around the world (Koo et al., 2016). In Southeast Asian countries, it is typically produced by fermenting fish, mixing fish and salt in a 3:1 ratio (Du et al., 2019; Lopecharat et al., 2001) and fermenting naturally for several months, or even longer periods of time. Fish sauce has not only unique flavors but also richer nutrients, including all essential amino acids, taurine vitamins, trace elements, and a large number of bioactive peptides (Lopecharat & Park, 2002), which are formed by decomposing the protein and fat of raw fish in the fermentation process with the combined action of proteases contained in the fish body, alongside microbial proteases (Giyatmi & Irianto, 2017).

Typically, in industry, starter culture is not used for the fermentation of fish sauce because the fermentation of fish sauce relies on native bacteria derived from raw materials. Due to the complexity of native strains, sometimes product quality is not homogenous and the fermentation time is often variable, both of which have been obstacles for the development of the fish sauce industry (Yongsawatdigul et al., 2007). The use of bacterial starter culture for fish sauce fermentation has been studied and shown to improve the flavor of fish sauce and to shorten the fermentation process (Fukami et al., 2004; Sun et al., 2016; Udomsil et al., 2017). It has been seen that both bacterial and fish proteinases gradually hydrolyze fish proteins during fermentation (Fukami et al., 2004; Giyatmi & Irianto, 2017). Bacteria break down the proteins and develop the aroma and flavor so highly desired, but when fish sauce is fermented under sterile conditions it was seen that the typical characteristic fish sauce aroma did not develop (Lopetcharat et al., 2001).

The feasibility of using *Tetragenococcus halophilus* and *Virgibacillus* sp. SK37, halophilic bacteria isolated from fish fermentation broth, as starter cultures for the flavor improvement of anchovy fish sauce fermentation has been previously studied (Udomsil, Chen, Rodtong, & Yongsawatdigul, 2017). The addition of both starter cultures in sequential order showed the potential to improve quality in terms of volatile compounds, glutamic acid content, and overall acceptability with a reduced level of biogenic amine content in the final product (ibid). Recently, it was reported that compared with spontaneous fermentation, products inoculated with starter cultures not only had the typical characteristics of fish sauce, but had better flavor and product quality in saltless (Gao et al., 2019) and low‐salt (3–6%, w/v) fish sauce (Bao et al., 2018; Zang et al., 2020; Zeng et al., 2017).

The use of starter culture could solve the problems associated with traditional production methods of fish sauces while also improving fish sauce quality. However, the development of starter culture for fish sauce fermentation where the salt concentration of the initial fermentation broth was close to 30% (w/v) seems to have been slow and generally unsuccessful.

The objective of this project was the screening and selection of halophilic microorganisms for use as starter cultures for the enhancement of the flavor of fish sauces with very high initial salt concentrations.

## 2 MATERIALS AND METHODS

### 2.1 Collection of samples to isolate halophilic bacteria

Fish broths were collected from fish sauce fermentation tanks in Phu Quoc (PQ), Nha Trang, and Phan Thiet, Vietnam. Fish exudates (FEs) and solar salt crystal were also collected from those factories. Seawater and mud were collected from ponds of salt pans in Vietnam. A fish fermentation broth was collected at the 20–25 cm depth from the top of fermentation tanks using a 10 ml disposable pipette and then mixed with the same volume of fish broth drained from the bottom of the same fish fermentation tank, with the mixture being used for the isolation of bacteria. All the samples were stored in a sterile container at 4°C for further examination.

### 2.2 Isolation of bacteria

Protease‐producing bacteria were isolated using JCM no. 377 agar (Booncharoen et al., 2019) with skim milk (JCMAS) containing 18% and 25% (w/v) NaCl applying the spread plate technique. Lactic acid bacteria (LAB) were isolated using De Man, Rogosa, and Sharpe agar (MRSA) plates containing 5‐–18% (w/v) NaCl and 0.5% (w/v) CaCO_3_. The fermentation broths from collection sites were diluted 10^0^, 10^1^, and 10^2^ times with 18% (w/v) NaCl and 0.1 ml of each dilute was plated onto JCMAS containing 18 and 25% (w/v) NaCl or MRSA containing various NaCl contents. Salt crystals of 1 g were mixed thoroughly with 1 ml of 18% (w/v) NaCl, diluted 10^0^, 10^1^, 10^2^ times with 18% (w/v) NaCl, and 0.1 ml of each dilute was plated, and incubated at 37°C for 7–10 days under aerobic conditions for the isolation of the protease strain, and 30°C for MRSA agar for 4–6 days in anaerobic conditions for LAB isolation using an anaerobic jar. A positive reaction for the proteolytic test and for LAB was indicated by way of a clear zone around the colony via hydrolyzing the skim milk and dissolving the turbid CaCO_3_ with acids produced by the LAB, respectively. The colonies showing clear zones around them were isolated and transferred by streaking onto agar plates for subsequent experiments.

### 2.3 Growth test on fish broth agar and fish exudate agar

To prepare fish broth agar (FBA), one part frozen anchovy was blended with two parts DW and the mixture was boiled for 25 min. The resulting fish slurry was filtered with cheesecloth. NaCl (18 and 25%, w/v), and 2% (w/v) agar were added to the filtrate. The pH was adjusted to 7.0, and the medium was autoclaved. To make fish exudate agar (FEA), FE was appropriately diluted with DW and mixed with 2% (w/v) agar. A culture isolate was inoculated using a toothpick onto the FBA or FEA and incubated at 37°C for 7–10 days aerobically and at 30°C for 5 days anaerobically for LAB, and their relative growth rates were recorded.

### 2.4 Sensory evaluation

In the early phase of screening project, isolated strains were screened for strains producing good fish sauce flavor by a sensory test employing 10 panelists who were fish sauce experts and had more than 10 years of experience in sensory evaluation of fish sauce and seasoning in Masan Industrial One Member Co, Ltd. For screening, one loopful of each selected isolate grown on JCMSA or MRSA (for LAB) was inoculated into a 10‐ml cap tube containing 8 ml fish broth fermented for 3 months (3 M fermented broth) (29.3–29.7% [w/v] NaCl, pH 5.58–5.90) in a fish fermentation tank in a Masan factory, and incubated at 30°C for 3 weeks. The control sample (without inoculated bacteria) and raw fish sauce from a factory were prepared for comparison. The aroma of samples was assessed in 3 steps. The first step was difference‐from‐control evaluation (negative control, sample without bacteria; positive control, nine‐month raw fish sauce from Masan factory) based on fish sauce‐like aroma using a 10‐point grading scale. The second step was In/Out assessment in which samples with high score in step 1 were evaluated for typical fish sauce characteristics (IN: possessed typical fish sauce notes; OUT: did not possessed typical fish sauce notes). Top nine samples that were assessed as IN by at least 50% of the panelists were chosen for descriptive analysis (step 3) based on a list of descriptors which was established by fish sauce experts worked for 10 years of production and also sent to flavor house of Takasago Singapore Pte. Ltd for GC‐MS analyses of volatile compounds.

Also, during the later validation tests of the selected isolates in glass fermentation jars for six months, the filtrates of the anchovy mash were evaluated for sensory qualities using the fish sauce experts as described above. Color, taste, and aroma of samples were evaluated on a 10‐point scale in which the higher the grade was the more similar to the nine‐month fermented fish sauce. Furthermore, panelists chose the top 3 samples which had the most fish sauce identity among the seven validating samples.

### 2.5 Volatile compound analysis using GC‐MS

The volatile compounds were assayed using the solid phase micro‐extraction GC‐MS method. The GC/MS was Agilent 7890A GC with 5977A MS (Align Technology Inc.). For the solid phase micro‐extraction method, a 4 g sample was extracted at 60°C for 45 min with no solvent. The SPME fiber used was SUPELCO 85 μm Carboxen/PDMS StableFlex.

### 2.6 Identification of bacteria

Sequencing of the 16S ribosomal RNA gene was used for bacterial identification. The phylogenetic tree was constructed using the neighbor‐joining method (Saitou & Nei, 1987). The optimal tree with the sum of branch length = 0.56849963 was presented. The percentage of replicate trees in which the associated taxa clustered together in the bootstrap test (1,000 replicates) were exhibited next to the branches (Felsenstein, 1985). The tree was drawn to scale, with branch lengths in the same units as those of the evolutionary distances used to deduce the phylogenetic tree. The evolutionary distances were calculated using the maximum composite likelihood method (Tamura et al., 2004), that being the units of the number of base substitutions per site. The analysis involved 46 nucleotide sequences. All ambiguous positions were removed for each sequence pair (pairwise deletion option). There were a total of 1,356 positions in the final dataset. Evolutionary analyses were performed in MEGA X (Kumar et al., 2016).

### 2.7 Growth characteristics and protease production of a selected isolate

Using FEA and JCMSA, optimal culture conditions were examined for cell growth and protease production, respectively. Various salt concentrations (10, 14, 18, 22, 26, w/v) in FE were prepared by diluting fish exudates from Masan PQ Corp (MPQ), Vietnam, containing 27% (w/v) salt with DW. Protease production was examined via a halo test using JCMSA containing different concentrations of MPQ sea salt. The optimal salt concentration was examined with different salt contents at 37°C and pH 7. Various culture temperatures (27, 32, 37, 42, 47°C) were used to determine the optimal culture temperature at pH 7 using 18% (w/v) salt. Various pHs (5.5, 6.0, 6.5. 7.0, 7.5) were also tested to determine the optimal growth pH at 37°C using 18% (w/v) salt. All the plates were incubated for 6 days, and their relative growth and halo sizes (cm) were recorded.

### 2.8 Validation of selected strains in fish fermentation

#### 2.8.1 Preparation of inoculum

One loopful of cells was dispersed in a well in 1 ml 18% (w/v) NaCl, pH 7.5, and inoculated into 100 ml of autoclaved fish exudate (FE) (obtained from Masan company; NaCl 29.5%, w/v) dilute containing 20% (w/v) NaCl in a 500 ml Erlenmeyer flask. Each flask was incubated at 37°C in a rotary shaking incubator operated at 200 rpm for 3 days. The flask containing FE inoculated with LAB strain was incubated at 37°C in a static condition for 3 days. The FE culture broth (8 ml) was inoculated into autoclaved FE broth (92 ml), pH 7.5, containing 20% (w/v) NaCl in a 250 ml Erlenmeyer flask and was incubated at 37°C in a rotary shaking incubator operated at 200 rpm (for LAB static condition) for 4 days. Before the usage of the 4 days cultured FE broth as an inoculum, the NaCl content of each culture broth was adjusted to 29.5% (w/v) by adding sea salt.

#### 2.8.2 Fish fermentation

The fermentation and inoculation were conducted simultaneously at time 0. One hundred milliliters of bacterial culture was mixed well with 500 ml FE in a 1‐L Erlenmeyer flask, and the mixture was mixed well again with 2 kg salted MPQ anchovy in a plastic container (38 cm top D, 32 cm bottom D, 39 cm H) using plastic‐gloved hands. For the control, 100 ml FE was used instead of bacterial culture. The entire mixture was transferred into a glass jar (22 cm L × 22 cm W × 35 cm H) with a metal cap. Fermentation was maintained at 30°C.

After 3 and 6 months, the fish mash (350 g) was taken from the glass jar using a sterilized big spoon and filtered through Whatman No. 1 filter paper. The filtrates (40 ml) were assayed for total nitrogen (TN), ammoniacal nitrogen (NH_3_‐N), amino nitrogen (AN), histamine, color, pH, amino acid profile, and sensory evaluation.

### 2.9 Analysis of TN, NH_3_‐N, AN, salinity, and cell number

TN is the total nitrogen content in the fish sauce and is used as an indicator for fish sauce classification (CODEX STAN 302–2011). NH_3_‐N is also known as rotten nitrogen, and the more fish sauce has, the less quality it becomes. AN is the nitrogen content in the form of amino acids, which determines the nutritional value of fish sauce.

TN content was determined by the Kjeldahl method (Sun et al., 2016). Ammonium in 5–20 ml samples after dilution was converted into ammonia during distillation. The amount of ammonia present, and thus the amount of NH_3_‐N present in the sample, was determined by back titration. The end of the condenser was dipped into 0.1 N sulfuric acid. The ammonia reacted with the acid and the remainder of the acid was then titrated with 0.1 N NaOH in the presence of methyl orange (0.1%, w/v) pH indicator. Program settings for the Kjeldahl distillation machine were 410 s, in which 3 s were for adding DW, 5 s for the reaction, and 402 s for distillation.

For AN analysis, 5–10 ml samples, with appropriate dilution, were adjusted to pH 10 using 0.1 N NaOH (thymolphthalein as pH indicator). Then, acid amines were converted into a salt form with copper by the addition of 25 ml 0.2 M copper (II) phosphate in borate buffer. Excess copper phosphate was removed by filtration. Ten milliliter clear filtrate was mixed well with 5 drops of concentrated acetic acid and 2 g KI. Ion Cu^2+^ reacted with ion I^‐^, resulting in I_2_ for which the concentration involved titration using 0.01 N Na_2_S_2_O_3_ in the presence of 1% starch indicator. From the difference in the amounts of 0.01 N Na_2_S_2_O_3_ used in samples with amino acid and controls (no amino acid), nitrogen content in amino acid was calculated.

Salinity was measured by using a salinity tester (Model Eutech Salt 6+, Thermo Fisher Scientific Inc.).

The bacterial number was determined using the standard plate count method. After shaking the glass bottle several times in a bottom up and down motion, fermented fish mash was removed from 3 different sites, at quantities of 2 ml from each site using a micropipette. The 6 ml samples were vortexed, and 100 μl was added into 900 μl of 18% (w/v) NaCl dilution solution and mixed thoroughly. After serial dilution to 10^–7^, 100 μl cell suspensions were plated on JCMSA medium containing 18% (w/v) NaCl. The plates were incubated at 37°C for 7–10 days, and colonies exhibiting the yellow color of *M. halotolerans* were enumerated.

### 2.10 Determination of histamine by HPLC

Biogenic amines in the fish sauce samples were extracted by protein precipitation in which 5 g of sample was precipitated for 10 min with 98% (v/v) absolute ethanol (Merck) and then centrifuged at 492 x*g*  for 10 min. After decanting and evaporating the supernatant, the samples were dissolved with 0.1 N HCl and then filtered through a 0.45‐μm filter unit before being injected into the chromatographic system for HPLC‐UV (Hitachi D‐7000). HPLC was performed with a C‐18 column (250 × 4.6 mm) as the stationary phase with a solution of 0.2 M phosphate buffer (pH 3.0)–acetonitrile–water (1:24:166, v/v) containing 2 mM sodium 1‐octane sulfonic acid as the mobile phase. A total flow rate of 1 ml/min was used, and the column thermostat was set at 30°C. Histamine content was quantified based upon peak areas using an appropriate standard curve.

### 2.11 Amino acid analysis by HPLC

Seventeen amino acids in fish sauce were extracted by acid hydrolysis before being injected into the HPLC system, by methods mentioned in Llames and Fontaine (1994). In brief, for the extraction of all amino acids except for cysteine and methionine, samples were hydrolyzed in 6 N HCl at 110°C for 24 hr in an evacuated sealed ampoule. For cysteine and methionine, prior to acid hydrolysis, samples were oxidized by performic acid reagent for 16 hr in order to be converted to cysteic acid and methionine sulfone. Then, sodium metabisulfite was added to remove excess performic acid. Extracted samples containing amino acid components were filtered before being injected in an amino acid analyzer system (AAA‐Hitachi‐L8800). The solvent system was sodium citrate/ethanol eluent with the elution being conducted in gradient mode for 55 min, with a flow rate of 0.4–0.5 ml/min.

## 3 RESULTS AND DISCUSSION

### 3.1 Isolation and selection of halophilic bacteria

Using JCMAS containing 18 and 25% (w/v) NaCl, a total of 148 strains were isolated from fish broths in fermentation tanks and FE, from several fish sauce factories and from salt crystals and salt pans. Among these latter, 100 strains were protease‐positive and 48 strains were protease‐negative, as judged by halo formation on JCMSA. From the 100 protease‐positive strains and 48 protease‐negative strains, 46 and 25 strains, respectively, which possessed the ability to grow on FBA containing 25% (w/v) NaCl, were selected.

In total, 196 strains exhibiting clear zones around their colonies by the acids produced on MRSA were also isolated. These strains were assumed to be LAB. Among these 196 strains, 50 with the ability to grow on MRSA containing 25% (w/v) NaCl were selected.

**Figure 1:**
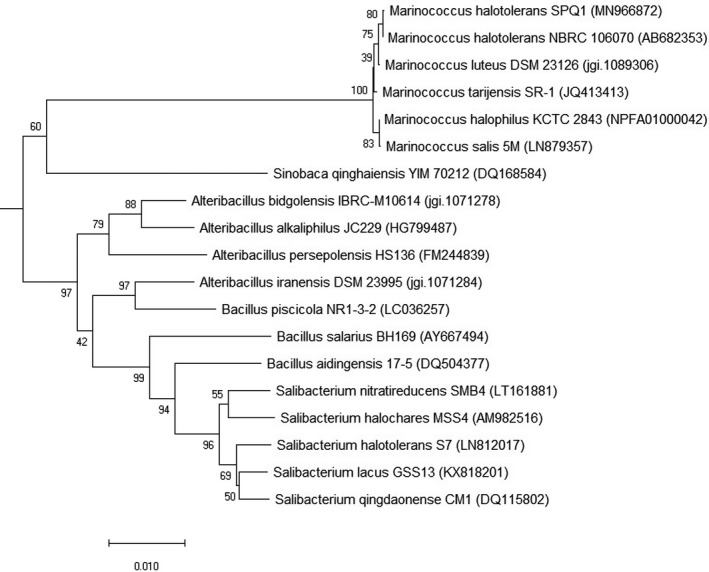
Neighbor‐joining tree showing the phylogeneitic position of an isolate SPQ, *Marinococcus* species, and related taxa based on 16S rRNA gene sequences. Numbers on branch nodes are bootstrap values (1,000 resampling). The scale bar refers to a phylogenetic distance of 0.01 substitutions per site

The three groups (protease‐positive, protease‐negative, and LAB) of strains capable of growing on 25% (w/v) NaCl were subjected to aroma evaluation. In the 3 M fermented broth for inoculation, having 29.3%–29.7% (w/v) NaCl and pH 5.58–5.90, the bacterial cells did not seem to multiply. However, many isolates exhibited a different, and often improved, aroma when compared with the control, indicating the cells were still functional for aroma formation. Two isolates from the 46 protease‐positive strains, 4 isolates from the 25 protease‐negative, and 3 isolates from the 50 LAB strains, with high aroma scores in the range of 50–70%, were selected and are illustrated in Table 1. These top 9 aroma isolates were then identified with 16S rRNA gene sequencing, revealing them to be in possession of 6 different names, as all 3 LAB isolates selected were *Tetragenococcus muriaticus*. Phylogenetic tree analysis was performed for the isolate SPQ, which had the top aroma score of 70%, indicating it falls within the cluster of *Marinococcus halotolerans* NRBC 106070 with 100.0% similarity (Figure 1). The *M. halotolerans* SPQ was isolated from a salt crystal in Masan PQ, Vietnam. *M. halotolerans* SPQ was finally selected for use in further investigation in this study.

**Table 1:** Selected top 9 aroma strains and their aroma profiles

Strain code	Identified scientific name	Percentage of panelists’ choices	Aroma profile
SPQ	*Marinococcus halotolerans*	70	Well‐fermented fish like, meaty, briny, heat‐like, sweet, fatty
M−51	*Tetragenococcus muriaticus*	60	Salted egg, rancid, over‐ripen fruit, fatty, heat‐like, meaty, fishy
6MR	*Tetragenococcus muriaticus*	60	Fermented fish like, briny, fishy from cook fish, vinegary
KSP	*Salicola salis*	60	Salted egg, briny, cheesy, smoky, fishy from dried fish
19‐J15	*Alkalibacillus almallahensis*	50	Fishy from cooked fish, fatty, briny
19‐U2	*Alkalibacillus almallahensis*	50	Fermented fish like, salted egg, meaty, heat‐like
63‐J15	*Bacillus aidingensis*	50	Smoky, sour‐aromatics, salted egg, fruity, alcoholic, burnt
H‐J21	*Halanerobium praevalens*	50	Heat‐like, briny, fishy from cooked fish
39‐R	*Tetragenococcus muriaticus*	50	Fermented fish like, heat‐like, meaty, briny, fatty, salted egg, animalic.

### 3.2 Growth characteristics and halo formation of *M. halotolerans* SPQ

**Figure 2:**
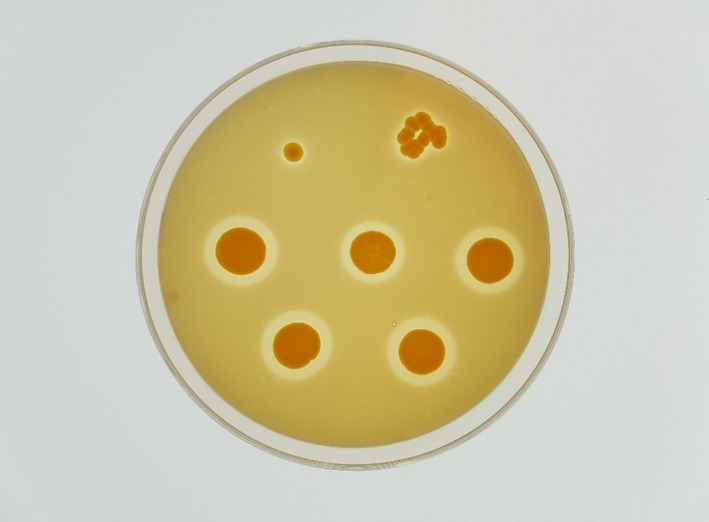
Halo–clear zone formation around the cell mass of *M. halotolerans* SPQ on a JCMSA plate containing 13% (w/v) NaCl

The color of cell mass grown on JCMSA was yellow (Figure 2). Cells were seen to grow well on FEA and JCMA at salt concentrations of 10–26% (w/v), at 27–47°C, and at pH 5.5–7.5. The range of conditions of halo formation on JCMA was narrower than those of growth, with the former being 10–14% NaCl (w/v), at 37–42°C, and at pH 7.0–7.5. The cells grew well on FEA in all the conditions tested, with this fast growth being an advantage in the preparation of inoculum for fish fermentation.

When *M. halotolerans* SPQ was isolated by JCMA containing 25% (w/v) NaCl, the halo–clear zone was absent around its colony and grouped into the protease‐negative strain. However, when the cells were cultured on 13% (w/v) NaCl, the halo formed around the cell mass.

### 3.3 Volatile compound analysis of *M. halotolerans* SPQ by GC‐MS

Table 2 shows the GC‐MS results of the fish broth fermented by *M. halotolerans* SPQ. Among the various volatile compounds analyzed, the concentration of several compounds such as ethyl alcohol, 1‐propanol, 1‐butyl alcohol, 1‐amyl alcohol, and methionol was significantly increased by the selected top flavor strain. Both samples formed short‐chain fatty acids, in which sum of volatile acids contents in control was 93.78%, higher than that (87.54%) of *M. halotolerans* SPQ. Volatile acids can be produced via lipolysis of fatty acid or amino acid metabolism. In turn, volatile acids are further oxidized resulting in alcohol, aldehyde, and ketone (Montel et al., 1998). It seems that addition of *M. halotolerans* SPQ facilitates oxidization reactions from acids, resulting in formation of more alcohols. *M. halotolerans* SPQ was still able to form these volatile compounds in harsh conditions of 29.3–29.7% (w/v) NaCl, at pH 5.58–5.90 in the 3 M fermented fish broth.

**Table 2:** GC‐MS results of the fish broth fermented by *M. halotolerans* SPQ

Component	Notes	Area %	
		Control	*M. halotolerans* MPQ
Ethyl acetate	Fruity	1.70	1.78
Ethyl alcohol	Winey	2.46	6.11
Methyl propyl ketone	Winey fermented	0.15	0.17
Butan−2‐ol	Winey	0.00	0.07
1‐Propanol	Winey	0.16	0.54
1‐Butyl alcohol	Winey	0.00	0.21
1‐Amyl alcohol	Fruity	0.09	0.99
Acetic acid	Vinegar	3.17	2.71
Methional	Sulfurous	0.07	0.07
Propionic acid	Fermented	3.84	3.20
1‐Butyric acid	Cheesy	5.79	5.28
Butyric acid	Cheesy	47.84	42.47
Furfuryl alcohol	Brown	0.12	0.21
1‐Valeric acid + 2‐Methylbutyric acid	Cheesy	18.22	18.49
Methionol	Sulfurous	0.29	0.96
Valeric acid	Cheesy	2.19	2.17
1‐Hexanoic acid	Cheesy	7.40	7.86
Caproic acid	Cheesy	0.23	0.25
Phenyl ethyl alcohol	Fermented brown	0.13	0.20
Phenol	Phenolic	0.51	0.56
Caprylic acid	Fatty	0.20	0.21
3‐(Methylthio)‐propionic acid	NA	Trace	Trace
Sulfurol	Creamy	Trace	Trace
4‐(Methylthio)‐butyric acid	NA	0.28	0.27
Phenylacetic acid	Sweet honey	0.56	0.49
3‐Phenylpropionic acid	Sweet honey	4.26	4.35

Abbreviation: NA, not applicable.

3‐(Methylthio)‐1‐propanol (methionol) possesses similar aromas to cauliflower, and cabbage is very important for the overall aroma of soy sauce and cheese (Yvon & Rijnen, 2001). 1‐Butyl alcohol also has a fragrant and pungent odor and is found in soy sauce and fish sauce (Giri et al., 2010). *Staphylococcus xylosus* R4Nu isolated from fish sauce mash increased 3‐methylbutanoic acid, 3‐methyl‐1‐butanol, and 2,6‐dimethylpyrazine in Thai fish sauce (Fukami et al., 2004). The use of starter cultures of *Virgibacillus* sp. and *Staphylococcus* sp. producing protease increased the desirable volatile compounds of fish sauce (Yongsawatdigul et al., 2007).

### 3.4 Validation test with anchovy inoculated with *M. halotolerans* SPQ

#### 3.4.1 TN, NH_3_‐N, AN, salinity, pH, and cell number in fish fermentation broth

**Figure 3:**
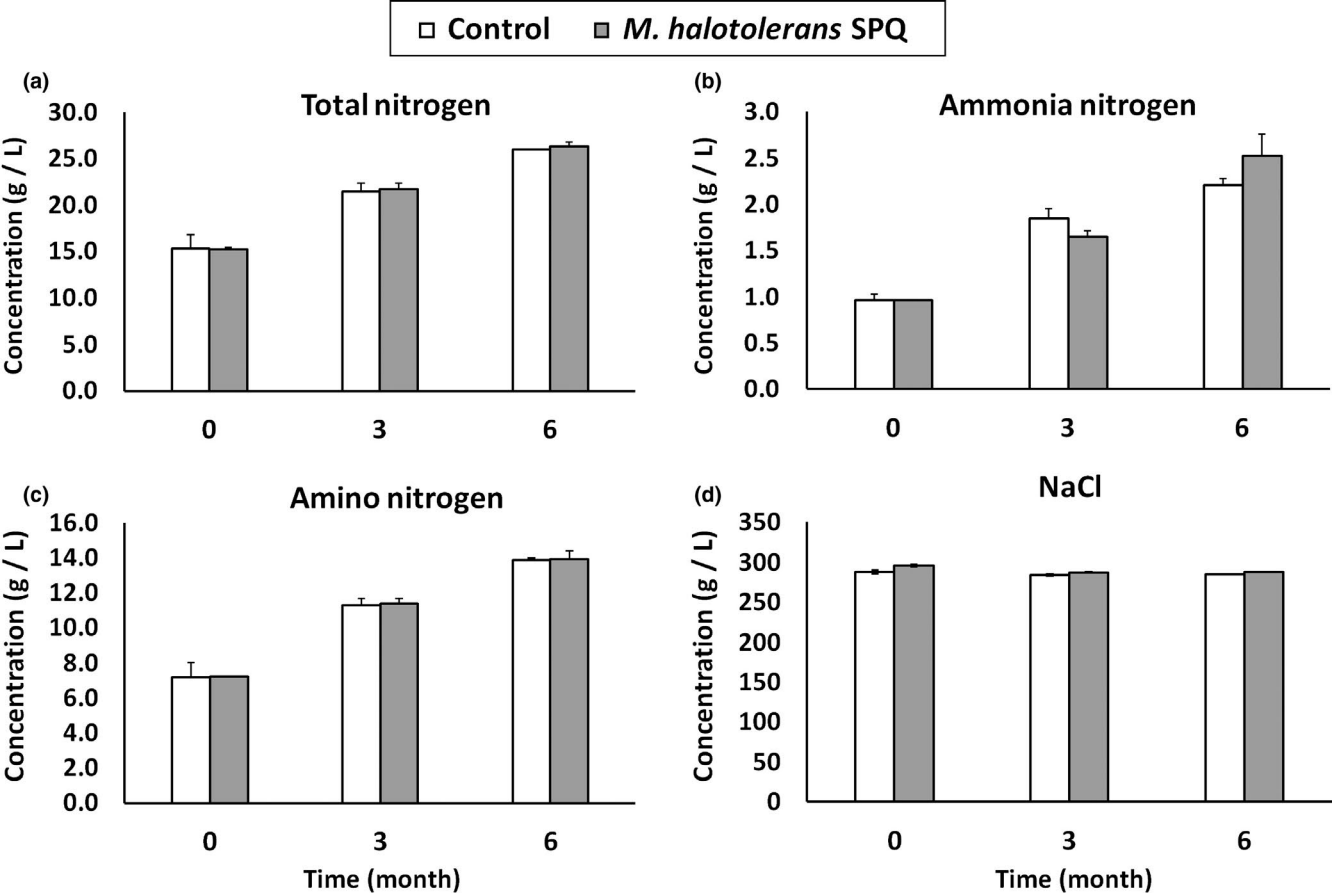
The change of TN, NH_3_‐N, AN, and NaCl contents in the fish fermentation broth with, or without, inoculum of *M. halotolerans* SPQ

Figure 3 shows the TN, NH_3_‐N, and AN values in an anchovy fermentation broth inoculated with *M. halotolerans* MPQ at the 3rd and 6th months. The TN and AN values in the inoculated broths increased with time, and these values were comparable to those of the control. The TN value represented the contents of the free amino acids, peptides, nucleotides, ammonia, urea, and trimethylamine in fish sauce (Udomsil et al., 2017). The NH_3_‐*N* value (2.52 g/L) in the inoculated broth at the 6th month was higher than that (2.21 g/L) of the control. The salinity of inoculated broth at the 3rd and 6th month was 287.3 and 287.5 g/L, respectively, and comparable to those of the control. It has also previously been reported that the NaCl concentration of fish sauce with starter cultures was comparable with those of the control, with the range being 27–28% (Udomsil et al., 2017). Since the Vietnamese standards for physical parameters in fish sauce (TCVN 5107:2018 Fish sauce) after fermentation are ≥ 10 g/L TN, ≥35% of TN/AN, ≤30% of NH_3_‐N/TN, and ≥ 245 g/L of NaCl, the physical parameters of fish broth fermented by *M. halotolerans* MPQ met the required standards of Vietnamese fish sauces.

The pH of the inoculated broth remained almost unchanged with time, being 5.78 at the beginning and 5.82 at 6 months, while the control's pH decreased, being 5.76 at the beginning and 5.43 at the 6th month. During fermentation, pH of fish sauce is slightly changed due to the release of free amino acid from protein and polypeptides, ammonium formation, and interconversions between amino acids and different compounds (Lopetcharat et al., 2011; Montel et al., 1998). As analyzed in Table 2, the total volatile content in the fish broth of *M. halotolerans* SPQ was lower than that control. It seems that addition of *M. halotolerans* SPQ accelerated the oxidization reactions of acids, resulting in reduced acid content. These reactions were taken place in flavor developing stage of fish fermentation from 4th month and therefore resulting in slightly increased pH of fish broth of *M. halotolerans*.

The inoculated cell numbers 6.5 Log CFU/ml of *M. halotolerans* MPQ in the fish fermentation broth decreased steadily to 2.0 Log CFU/ml at 3 months and finally reached zero at 6 months. Udomsil also reported that 6.39 Log CFU/ml of *Virgibacillus* sp. SK37 had reduced to 1.3 Log CFU/ml at 3 months and to zero at 6 months (ibid).

#### 3.4.2 Histamine content in fish fermentation broth

One of the most common forms of intoxication is histamine poisoning caused by the ingestion of fish and fishery products. High contents of histamine in foods can have important vasoactive effects in humans (Lehane & Olley, 2000). Histamine is produced in fish tissue through the decarboxylation of free histidine by exogenous decarboxylases released by microorganisms. Exogenous decarboxylases released by microorganisms decarboxylates‐free histidine, and histamine is thus produced in fish tissue. European Legislation has set the regulatory limit of histamine at a maximum of 200 mg/kg in fresh fish (Visciano et al., 2014).

The histamine content of fish broth inoculated with *M. halotolerans* SPQ after 3 and 6 months was 147.82 and 110.12 mg/L, respectively, while those of the control were 140.54 and 106.63 mg/L, respectively. The histamine content in both samples significantly decreased at 6th month compared with at 3rd month. The observed histamine content was lower than the 200 mg/L standard level limit, indicating that fermented fish broth inoculated with *M. halotolerans* SPQ is safe. In a preliminary test, cells of *M. halotolerans* SPQ were inoculated into JCM containing 0.25% (w/v) histidine and cultured at 30℃ for 18 days, but histamine was not detected, whereas one of our several other isolates, *Tetragenococcus halophilus* 63–25‐R15‐1, produced 645.0 mg/L (data not shown). The above results indicate that *M. halotolerans* SPQ was not able to produce histamine.

#### 3.4.3 Amino acid profile in fish fermentation broth

The contents of various amino acids in the fish fermentation broth after 6 months are illustrated in Table 3. Aspartic acid, glutamic acid, and lysine were the predominant amino acids in both broths. These results were consistent with the amino acid profiles of other fish sauces (Park et al., 2001; Rabie et al., 2018; Yongsawatdigul et al., 2007). Stefansson et al. (1995) noted that during the ripening of salted herring, there was a gradual increase of either peptides or free amino acids, and these compounds are believed to play an important role in the organoleptic properties of end products. The free amino acids are precursors of flavor compounds as well as important flavor compounds (Ardo, 2006; Flores & Toldra, 2011).

**Table 3:** The amino acid profile in fish fermentation broth of *M. halotolerans* SPQ

Component	Composition (g/kg)		Taste (Kirimura, 1969)
	Control	SPQ	
Methionine	3.58 ± 0.50	2.64 ± 0.37	ND
Tryptophan	2.51 ± 0.38	2.59 ± 0.39	Bitter
Alanine	12.09 ± 1.82	9.48 ± 1.43	Sweet
Arginine	0.50 ± 0.06	0.99 ± 0.12	Bitter
Aspartic Acid	18.48 ± 2.65	22.83 ± 3.27	Umami
Cystine	0.67 ± 0.10	0.91 ± 0.14	ND
Glutamic Acid	23.02 ± 2.85	31.09 ± 3.85	Umami
Glycine	9.23 ± 1.31	10.62 ± 1.50	Sweet
Histidine	7.30 ± 1.03	7.06 ± 1.00	Bitter
Isoleucine	4.87 ± 0.60	4.40 ± 0.54	Bitter
Leucine	4.64 ± 0.53	5.70 ± 0.65	Bitter
Lysine	20.91 ± 4.46	18.78 ± 4.01	ND
Phenylalanine	5.87 ± 0.79	5.41 ± 0.73	Bitter
Proline	4.42 ± 0.64	4.88 ± 0.71	Sweet
Serine	9.69 ± 1.31	9.86 ± 1.33	Sweet
Threonine	11.17 ± 1.84	11.47 ± 1.89	Sweet
Tyrosine	1.33 ± 0.17	1.03 ± 0.14	ND
Valine	11.51 ± 1.59	10.69 ± 1.47	Bitter

Abbreviation: ND, not described.

Two important umami amino acids, aspartic acid and glutamic acid, increased by 23.5 and 35.1%, respectively, in the inoculated fermentation broth (Table 3). The action of exoproteinases, namely aminopeptidase and carboxypeptidases, is responsible for formation of free amino acids during fish sauce fermentation. In addition, *γ*‐glutamyltranspeptidases and glutaminases that catalyze the hydrolysis of *γ*‐glutamyl compounds to glutamic acid are important enzymes responsible for free glutamic formation and flavor enhancement during soy sauce fermentation (Weingand‐Ziade, Gerber‐Decombaz, Affolter M, & others, 2003). The enzymes were widely distributed in *Micrococcus luteus*, *Lactobacillus rhamnosus*, and various species of *Bacillus* (Nandakumar & others, 2003). These enzymes could be important for increasing free amino, particularly glutamic acid, in fish sauce fermentation. Aspartic acid is synthesized from oxaloacetate, an intermediate of the TCA cycle by a transamination reaction with L‐glutamate by aspartate aminotransferase.

The taste of umami, saltiness, sweetness, sourness, and bitterness was found in fermented aquatic products (Liu et al., 2017; Lopetcharat et al., 2001; Zhao et al., 2016), with the umami taste considered to be the most important taste (Hajeb & Jinap, 2015; Yoshida, 1998). Since the intensity of the umami taste of monosodium L‐glutamate is about 4 times higher than that of monosodium L‐aspartate (Kato et al., 1989), free L‐glutamic acid in the presence of salt is the essential umami taste forming substance.

In this study, extremely harsh salinity conditions of 28.7–29.7 (w/v)  NaCl combined with a low pH of 5.76–5.82 still allowed *M. halotolerans* SPQ to produce significantly more umami amino acids than the control. The inoculated cell numbers, determined by standard plate‐counting method, of 6.5 Log CFU/ml in the fish fermentation broth decreased progressively to 2.0 Log CFU/ml at 3 months and finally reached to zero at 6 months. However, according to Udomsil et al. (2017), the starter cultures therein did not decrease to zero, but remained in a *“viable but nonculturable stage,”* which could be detected using the molecular‐based technique of qPCR. According to Udomsil et al. (2017), the actual cell numbers assayed by qPCR were 1.5–2.0 Log CFU/ml higher than those determined by the plate‐counting method. Therefore, the viable cell number of the inoculated culture in the fermentation broth could be underestimated when using a culture‐dependent counting method. A viable, but nonculturable stage of bacteria, is thought to be a morphological adaptation that promotes long‐term survival under extreme conditions, such as high or low temperatures, rapid changes in pH or salinity, malnutrition, lack of oxygen, and osmotic pressure (Frankenhuyzen et al., 2013; Oliver, 2010).

#### 3.4.4 Sensory evaluation

Six strains were sensory‐evaluated after 6 months of fermentation, and the color, odor, and taste of fish sauce samples are presented in Table 4. The results show that *M. halotolerans* SPQ had the highest preference to the panelists among the selected strains. *M. halotolerans* SPQ had also the higher score of aroma and taste, compared to reference sample which was raw nine‐month fish sauce drawn from 13‐ton fermentation tank at Masan PQ factory and was also of preference to the panelists in terms of typical fish sauce identity among the selected strains. This result again confirmed that *Marinococcus* MPQ was good flavor strain among those examined.

**Table 4:** Results of sensory evaluation of the fish sauce fermented by selected isolates^a^

Strain code	Identified scientific name	Color	Aroma	Taste
	Reference sample^a^	7.0	6.2	7.2
SPQ	*Marinococcus halotolerans*	6.8	7.0	7.0
M−51	*Tetragenococcus muriaticus*	7.0	6.4	6.6
KSP	*Salicola salis*	6.4	6.0	5.4
19‐J15	*Alkalibacillus almallahensis*	6.8	6.4	6.2
63‐J15	*Bacillus aidingensis*	6.6	6.0	5.6
H‐J21	*Halanerobium praevalens*	6.0	6.2	5.8

^a^ The fish sauce was fermented at 30°C for 6 months.

^b^ Reference sample was a raw nine‐month fish sauce drawn from 13‐ton fermentation tank in Masan PQ factory.

Several other bacterial isolates have been elucidated for their potential as starter cultures for the improvement of fish sauce flavor. The cultures include *Staphylococcus xylosus* (Fukami et al., 2004), *Staphylococcus* sp. (Yongsawatdigul et al., 2007), *T. halophilus* (Udomsil et al., 2010, 2011), *Aspergillus oryzae* (Sun et al., 2016), *Halobacterium salinarum* (Alfonzo et al., 2017), *Lactobacillus lactis* and *Weissella cibaria,* and *L. plantarum* and *Saccharomyces cerevisiae* (Zang et al., 2020). In this study, we have added to the list demonstrating that *M*. *halotolerans* isolate is a promising flavor strain as a starter culture for fish sauce fermentation, especially for fish mash containing 30% (w/v) NaCl.

## 4 CONCLUSION

Traditionally, fish sauce has been produced by fermenting fish under natural conditions. The use of starter culture could improve the quality of fish sauces. Flavor is a particularly important indicator of consumer acceptance of fish sauce. The development of starter culture for the fermentation of fish sauce has not been particularly successful, especially when the salt concentration of the initial the fish fermentation broth was close to 30% (w/v). We have shown that *M*. *halotolerans* isolate is a promising flavor strain as a starter culture for fish sauce fermentation. This strain could also shorten the fermentation time by reaching a desirable level of flavor within a shorter time frame.

## CONFLICTS OF INTEREST

The authors have no conflicts interests to declare.

## AUTHOR CONTRIBUTIONS

K. Kim designed the study and interpreted the results. A. D. Q. Nguyen, A. Sekar, and N. T. Le collected test data and drafted the manuscript. M. Kim, L. P. Nguyen, S. Uh, and S. Hong conducted the experiments.
